# Naltrexone Reverses Ethanol-Induced Rat Hippocampal and Serum Oxidative Damage

**DOI:** 10.1155/2013/296898

**Published:** 2013-12-01

**Authors:** Inmaculada Almansa, Jorge M. Barcia, Rosa López-Pedrajas, María Muriach, María Miranda, Francisco Javier Romero

**Affiliations:** ^1^Departamento de Ciencias Biomédicas, Instituto de Ciencias Biomédicas, Universidad CEU Cardenal Herrera, Moncada, 46113 Valencia, Spain; ^2^Facultad de Medicina, Universidad Católica de Valencia “San Vicente Mártir”, C/ Quevedo 2, 46001 Valencia, Spain; ^3^Facultad de Ciencias de la Salud, Universitat Jaume I, 12071 Castellón, Spain

## Abstract

Naltrexone, an antagonist of **μ**-opioid receptors, is clinically used as adjuvant therapy of alcohol dishabituation. The aim of the present work was to test the effect of 1 mg/kg body weight of naltrexone to revert oxidative stress-related biochemical alterations, in the hippocampus and serum of chronic alcoholic adult rats. Malondialdehyde concentration was increased and glutathione peroxidase activity was decreased in hippocampus and serum of alcohol-treated rats. Naltrexone treatment restored these alterations. The in vitro antioxidant ability of Ntx could not justify these effects considering the doses used. Thus this apparent protective effect of Ntx can only be attributed to its pharmacological effects, as herein discussed.

## 1. Introduction

One of the goals of drug therapy for alcoholism is to avoid or reduce alcohol consumption and relapse. In this sense, opioid antagonists have been used for alcoholism treatment [1]. The use of these substances is based on their property to block opioid receptors and thereby reduce the reinforcing properties of alcohol and opioids. Naltrexone (Ntx) is the opioid antagonist most widely used for alcoholism because of its safety and effectiveness in reducing alcohol intake and craving and preventing relapse [2].

It has been proposed that endogenous opioids may modulate oxidative stress and therefore affect cell proliferation and survival [3]. In fact, several reports suggest that Ntx shows antiinflammatory and immunomodulatory properties [4]. Regarding oxidative stress and glutathione (GSH) metabolism, Ntx is able to normalize hepatic GSH and lipid peroxidation (LPO) levels while decreasing the number of apoptotic cells in a rat model of chronic cholestasis [3].

Free radicals may directly or indirectly affect several cellular and physiological mechanisms. They may lead to alterations in vital macromolecules of living cells, including DNA or membrane lipids. It has been shown that chronic alcohol consumption leads to an increase of lipid peroxidation products and a decrease of antioxidant factors such as GSH and related enzymes in the liver [5], brain [6], retina [7], and optic nerve [8].

The lipid content of the cellular membrane can be attacked by ROS, and thus LPO represents a typical marker of ROS action over lipidic membranes [9]. Central nervous system (CNS) is highly sensitive to oxidative stress because of its high oxygen consumption and lipid content, specially polyunsaturated fatty acids but with low antioxidant defense activity [10].

It has been previously reported that acute and chronic ethanol administration induces oxidative stress, producing LPO as well as modifying antioxidant enzyme activities in rat hippocampus and blood [11–13]. This work reports a novel finding on the “antioxidant-like” effects of Ntx after ethanol exposure.

## 2. Methods 

### 2.1. Experimental Design

Thirty-two male Sprague-Dawley (SD) rats weighing 306.58 ± 2.08 gr were housed in individual cages with a 7 a.m. to 7 p.m. dark-light cycle, with controlled temperature and humidity. All animal manipulations were done according to international regulations of the European Parliament and Council (2003/65/CE) and were approved by the animal care and use committee of the University Cardenal Herrera. Rats were randomly divided into two groups (*n* = 16 each) receiving either control or alcohol liquid diet [14]. Ethanol liquid diet was administered *ad libitum *along the duration of the treatment (6 weeks), and control animals received the same volume of the corresponding pair on the following day (pair-fed control). At the end of the 4th week of ethanol administration, ethanol and control groups were randomly assigned into two subgroups each (*n* = 8 as each group), receiving daily either Ntx (1 mg/Kg/i.p.) or saline (same volume Ntx i.p.), keeping the same ethanol or control liquid diet for two additional weeks.

Liquid ethanol and control diets were purchased from Test Diet (London, UK) and prepared in liquid form with either ethanol plus water (resulting in a 6.4% v/v ethanol concentration) or water alone. These diets have been developed to supply isocaloric intake in both alcoholic and nonalcoholic conditions, by supplementing the latter with dextrinated maltose accordingly (ethanol-derived calories at 6.4% ethanol concentration provide 350 kcal/L).

Body weight was initially and finally recorded, and ethanol blood level was randomly measured along the last 3 weeks of treatment by means of a standardized colorimetric assay kit (BIOLABO, Maizy, France). Blood samples were obtained from the tail.

All animals were sacrificed at the end of the experiment by overdose of sodium pentobarbital, then brains were rapidly extracted, and both hippocampi were dissected and homogenized in 0.2 M potassium phosphate buffer, pH 7.0. These homogenates were used to assay glutathione peroxidase (GPx) activity and protein and malondialdehyde (MDA) concentrations. Samples were kept frozen (−80°C) until biochemical assays were performed.

The blood samples were allowed to clot at room temperature and were centrifuged at 3,000 g for 10 min to separate the serum, which was stored at −20°C for later analysis.

### 2.2. Biochemical Assays: Oxidative Stress Markers and Protein Determination

Protein content was measured by means of the Lowry method [15]. Glutathione peroxidase activity, which catalyzes the oxidation by H_2_O_2_ of GSH to its disulfide (GSSG), was assayed spectrophotometrically as reported by Lawrence et al. [16] towards hydrogen peroxide, by monitoring the oxidation of NADPH at 340 nm. The reaction mixture consisted of 240 mU/mL of glutathione disulfide reductase, 1 mM GSH, 0.15 mM NADPH in 0.1 M potassium phosphate buffer, pH 7.0, containing 1 mM EDTA and 1 mM sodium azide; a 50 *μ*L sample was added to this mixture and allowed to equilibrate at 37°C for 3 min. Reaction was started by the addition of hydrogen peroxide to adjust the final volume of the assay mixture to 1 mL.

The concentration of malondialdehyde (MDA), a lipid peroxidation product, was measured by liquid chromatography as previously described [17]. Briefly, 0.1 mL of sample and 0.75 mL of working solution (thiobarbituric acid 0.37% and perchloric acid 6.4%, 2 : 1 v/v) were mixed and heated to 95°C for 1 h. After cooling (10 min in an ice water bath), the flocculent precipitate was removed by centrifugation at 12,000 rpm for 10 min. The supernatant (0.2 mL) was neutralised to pH 6-7 and filtered with a syringe filter prior to injection on a Cromasil C18 5 lm column (150 9 4.6 mm). The mobile phase consisted of 50 mM phosphate buffer (pH 6.0): methanol (58 : 42, v/v). Isocratic separation was performed with 1.0 mL/min flow (HPLC System, Waters) and detection at 532 nm (UV/VIS HPLC-Detector 2475, Waters). Calibration curves were run daily.

### 2.3. Total Antioxidant Capacity

Total antioxidant capacity of the naltrexone solutions was determined with the Antioxidant Assay Kit (Cayman Chemical Company). The assay was performed according to manufacturer's instructions. Absorbance was measured at 405 nm using a plate reader (VICTOR Perkin Elmer 2030).

### 2.4. Statistical Analysis

The results are presented as mean values ± SE. Statistical significance was assessed by Student's *t*-test (when comparing two groups) or when necessary by ANOVA followed by the least significant difference (LSD) test (when comparing more than two groups). The level of significance was set at *P* < 0.05.

## 3. Results 

The animals mean initial weight was 284 ± 26 g in control group, 299 ± 7 g in ethanol group, 277 ± 36 g in control plus naltrexone group, and 302 ± 6 g in ethanol plus naltrexone group. While at the time of sacrifice it was 398 ± 12 g in control group, 384 ± 77 g in ethanol group, 394 ± 19 g in control plus naltrexone group, and 383 ± 7 g in ethanol plus naltrexone group. There were no statistically significant differences in weight variation between groups in any phase of the experiment (*P* > 0.05).

Blood ethanol concentrations of alcoholic rats ranged from 8.6 to 20.2 mmol/L, which were similar to previous studies with this model and are clinically relevant [18].

The liquid diet volume consumed by rats receiving ethanol was 80 ± 15 mL/d or 78 ± 18 mL/d (mean ± SD) in control animals. Animals receiving ethanol plus Ntx consumed 78 ± 12 mL/d and the control + Ntx group consumed 76 ± 18 mL/d. No statistically significant differences were found among groups in terms of liquid diet volume consumption.

### 3.1. Hippocampal Oxidative Stress Markers and Naltrexone

**Figure 1:**
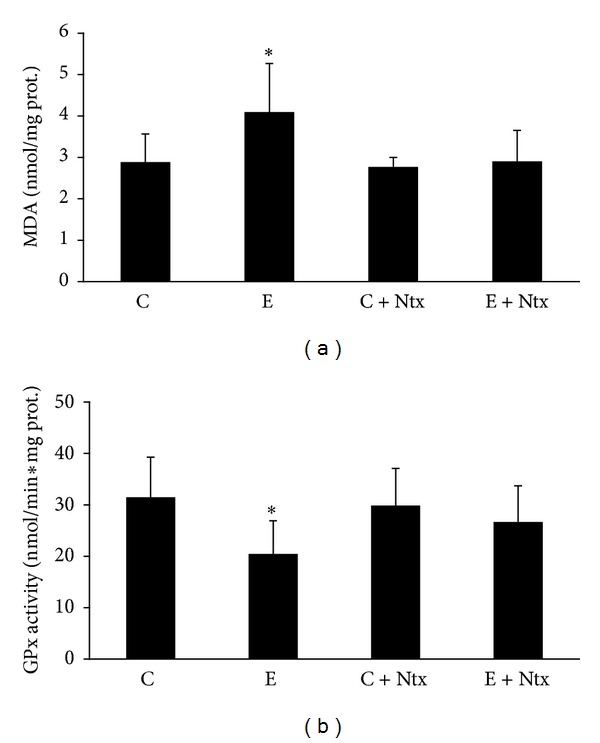
(a) MDA concentration in the hippocampus of animals from control (C), ethanol (E), control plus naltrexone (C + Ntx), and ethanol plus naltrexone (E + Ntx) groups. *P < 0.05 versus all groups. (b) Gpx activity in the hippocampus of animals from control (C), ethanol (E), control plus naltrexone (C + Ntx), and ethanol plus naltrexone (E + Ntx) groups. *P < 0.05 versus C and C + Ntx.

Six weeks of chronic ethanol consumption produced a statistically significantly increase of hippocampal MDA levels (Figure 1(a)). Ntx was able to reverse this hippocampal MDA increase. Ethanol treatment produced a statistically significant decrease of hippocampal GPx activity compared to that in control group (Figure 1(b)), whereas GPx activity in the Ntx-treated group could not recover completely to control values but showed a clear tendency.

### 3.2. Effects of Naltrexone on Systemic Oxidative Stress Markers

**Figure 2:**
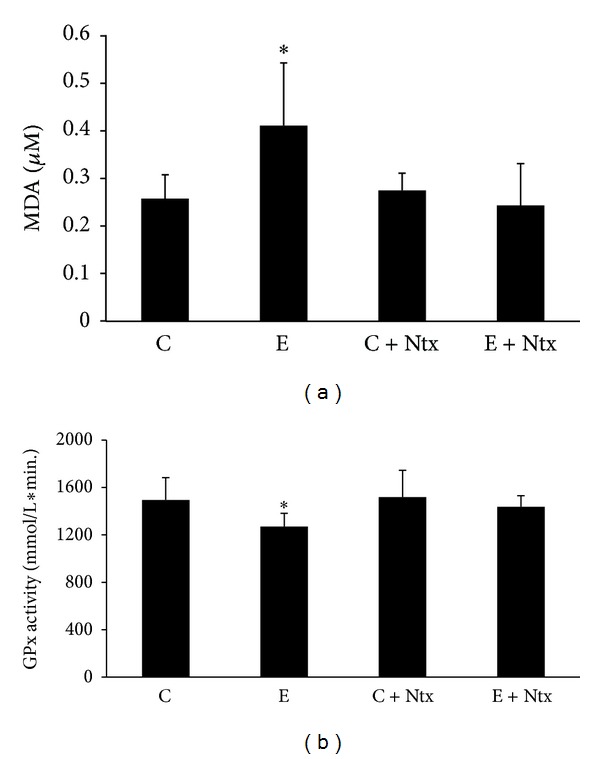
(a) MDA concentration in serum of animals from control (C), ethanol (E), control plus naltrexone (C + Ntx), and ethanol plus naltrexone (E + Ntx) groups. *P < 0.05 versus all groups. (b) Gpx activity in serum of animals from control (C), ethanol (E), control plus naltrexone (C + Ntx), and ethanol plus naltrexone (E + Ntx), groups. *P < 0.05 versus all groups.

Serum MDA level was statistically significantly increased only in the ethanol-treated group (Figure 2(a)), indicating that Ntx treatment restores MDA serum levels to control values.

A statistically significant decrease on serum GPx activity was observed in the ethanol-treated rats, and the administration of Ntx, on the last two weeks of ethanol intake, recovered serum Gpx activity (Figure 2(b)).

### 3.3. Antioxidant Capacity of Naltrexone

**Figure 3:**
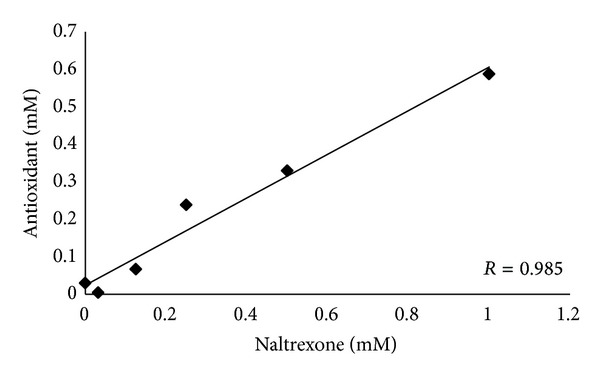
In vitro test of the antioxidant capacity of different concentrations of Ntx solution (*r* = 0.985; *P* < 0.001). The best fit curve corresponds to *y* = 0.582*x* + 0.0242.

In order to test whether these effects of Ntx could be attributed to direct antioxidant properties of Ntx, antioxidant capacity was assayed to test this possibility.  Figure 3 shows the linear correlation (*r* = 0.985; *P* < 0.001) between Ntx concentration and antioxidant capacity.

## 4. Discussion

Present data confirmed that chronic ethanol consumption causes LPO and therefore increases MDA levels, while it decreases GPx activity (Figures 1 and 2). This is consistent with previous findings describing that chronically administered ethanol induces oxidative stress in rat hippocampus and retina [7, 13].

Actually, different mechanisms of action dealing with oxidative stress have been proposed for Ntx. Several studies suggest that endogenous opioids modulate oxidative stress in different tissues affecting cell survival and proliferation [3]. Pretreatment with Ntx significantly reduced the circulatory failure and liver dysfunction in a rat model of induced sepsis by lipopolysaccharide (LPS). These effects are associated with reduced TNF-*α* levels and decreased superoxide anion formation, considering that Ntx was administered before LPS treatment [4]. Kiani and coworkers showed that Ntx decreased liver injury in rats and mice with acute biliary obstruction. Although the mechanisms by which Ntx ameliorates liver injury remain unknown, these authors suggested the prevention of hepatic GSH decrease by opioid receptors blockade as a plausible mechanism of action for Ntx [19], though other nonopioid effect(s) of Ntx could also be involved in their as well as our results. Unlike previous reports [4, 19], one differential point of the present work is that Ntx was administered after 4 weeks of ethanol consumption and not before ethanol treatment in a more preventive model. As reported herein, Ntx normalized MDA increased levels in both hippocampus and serum. However, Ntx recovered enzymatic Gpx activity to control values in serum and almost in hippocampal samples.

Although it is demonstrated that Ntx has by itself antioxidant capacity, this antioxidant effect is significant at concentrations in the mM range, whereas the theoretical concentration order optimally reached in plasma could be calculated around the *μ*M one. So it seems improbable that the observed effects on hippocampus and serum could be attributed to direct Ntx effects. Therefore, an opioid-mediated signalling mechanism might be responsible for the observed effects of Ntx, rather than a direct molecular effect, considering the Ntx doses used.

Chronic ethanol consumption modulates the expression of nitric oxide synthase (NOS), modulating NO production in several brain areas [20, 21]. The reduction of ethanol-induced MDA levels by Ntx could be explained by the NOS activity reduction that could result in NO decay and finally less peroxynitrite generation [22, 23]. Although little is known about the mechanisms of action by which Ntx works against alcoholism, present results provide evidence of additional benefits of Ntx in improving some ethanol-induced oxidative misbalance in rat serum and hippocampus.

## 5. Conclusion

Naltrexone at the dose used is able to reverse oxidative stress misbalance induced by chronic ethanol treatment in rat hippocampus and serum. More studies must be addressed to resolve the opened question related to opioid receptors and the antioxidant effect of Ntx.
